# Immunological effect of ten-year c-ART in treatment-naive and pre-treated HIV-1 patients in Bulgaria

**DOI:** 10.1186/1742-4690-9-S1-P12

**Published:** 2012-05-25

**Authors:** Ivaylo Elenkov, Maria Nikolova, Ivanka Radeva, Margarita Yankova, Nina Yancheva

**Affiliations:** 1 Specialized Hospital for Active Treatment of Infectious and Parasitic Diseases, Sofia, Bulgaria; 2 Clinical Immunology at National Center of Infectious and Parasitic Diseases, Sofia, Bulgaria

## Introduction

Highly effective combination anti-retroviral therapy (c-ART) has been applied in Bulgaria since 1999. The aim of the present study was to compare retrospectively the long-term immunological effect of c-ART in treatment-naïve and pre-treated HIV-1+ patients.

## Patients and methods

The study included HIV-1+ patients (n=56) that have started c-ART between March 1999 and December 2001, have been on continuous treatment, with good adherence, death being the only reason for ART stop. Of them, 27 had a history of irregular pre-treatment with AZT or AZT/LMV for an average of 4.6 yrs (Group A), and 29 were ART-naïve (Group B). CD4 absolute counts (AC) were determined by single-platform flow cytometry (BD Biosciences). Viral load (VL) was measured by RT-PCR (Roche). Comparisons were performed by unpaired t-test (SPSS 17.0).

## Results

The demographic characteristics of groups A and B did not differ significantly: mean age (yrs): 34 vs. 35; male to female ratio: 9 vs. 7, respectively. Baseline CD4 AC (cells/ml) and VL (log HIV RNA copies/ml) were comparable: mean 124 vs. 119, and 5.1 vs. 4.6, respectively, (p>0.05 for both comparisons). In the long term, suppression of viral replication was observed in both groups: mean VL at 5 yrs 3.7 vs. 3.1 for groups A and B, respectively, (p>0.05). However, treatment-naïve patients (group B) had a better immune recovery than group A, and the difference became significant in the long term: mean CD4 AC 177 vs. 252 after 6 months of c-ART, (p>0.05), 391 vs. 240 at 2 yrs (p

## Conclusion

Similarly to other studies, (SHM Monitoring report, 2009), a more complete and lasting long-term immunologic response to c-ART was observed in treatment – naïve patients. According to us, a previous sub-optimal and irregularly applied ART regimen, may promote the selection of gradually outgrowing drug-resistant viral strains, compromising therapeutic efficacy in the long run.

